# Ophthalmic Care Utilization and Out-of-Pocket Expenditure in Iran: Kurdistan Eye Health and Economics Survey-2015

**Published:** 2019-07

**Authors:** Seyed-Farzad MOHAMMADI, Cyrus ALINIA, Ebrahim GHADERI, Alireza LASHAY, Mahmoud JABBARVAND, Elham ASHRAFI, Naser NOURMOHAMMADI, Saeid SHAHRAZ

**Affiliations:** 1. Translational Ophthalmology Research Center, Farabi Eye Hospital, Tehran University of Medical Sciences, Tehran, Iran; 2. Department of Public Health, Urmia University of Medical Sciences, Urmia, Iran; 3. Social Determinants of Health Research Center, Research Institute for Health Development, Kurdistan University of Medical Sciences, Sanandaj, Iran; 4. Tufts Medical Center, Institute for Clinical Research and Health Policy Studies, Boston, Massachusetts, USA

**Keywords:** Ophthalmic services, Utilization, Out-of-pocket, Heckman two-step regression model, Social determinants of health (SDH)

## Abstract

**Background:**

Due to lack of information about ophthalmic economics in Iran, health policy makers unable to distribute resources optimally in terms of efficiency and equity. Therefore, we estimated the total and eye care utilization, out-of-pocket expenditures, and its association with social determinants of health in Iran in 2015.

**Methods:**

A multi-stage population-based, cross-sectional study in a random sample aged 50 yr or older in Kurdistan Province, Northwest Iran was used. The utilization rate of eye and general health care and related out-of-pocket expenditures was estimated during the recent last six months. To find the association between social factors and care out-of-pocket expenditures, we used a Heckman two-step regression model.

**Results:**

About 81% and 37% of participants were utilized the health and ophthalmic services, respectively. Statistically significant lower ophthalmic utilization rates were observed among men, middle-aged population, illiterate participants, rural residents, daily-paid workers, and the poorest participants. The average of vision and total health-related out-of-pocket expenditures among those used these services have estimated as US$43.7 (SE: 2.6) and US$439.9 (SE: 22.8), respectively. The highest (US$ 396.6) and lowest (US$ 10.4) ophthalmic out-of-pocket costs were related to patients with Glaucoma and Central Nervous System abnormalities, respectively. Multivariate analyses confirmed an unequal probability of having the ophthalmic out-of-pocket expenditures among different subgroups especially in favor of females, older, and those with more severe visual impairment.

**Conclusion:**

Ophthalmic disorders reconstituted about 10% of all health services OOP expenditures on average among individuals older than 50 yr.

## Introduction

Although the most eye problems and their caused blindness are avoidable (1) but the burden of eye disorders has considerably increased, over the past two decades in Iran (2). While elderly are subject to a considerable burden of vision disorders, barriers exist to the utilization of regular eye services and corrective interventions (3). One concern is that the use of Out of Pocket (OOP) may lead to suboptimal use of essential eye services and medications, particularly among the poor (4). The degree of cost sharing between individuals and third-party payer affects preventive service use (5). There is evidence that confirmed the existing of a considerable unmet need for ophthalmic services in Iran (6–8).

WHO’s VISION 2020 is an international initiative aiming to eliminate avoidable blindness by 2020 (9) emphasized on overcoming the barriers in utilizing the ophthalmic services and increasing the equality in receiving these services (10). Nonetheless, few studies have investigated the financial access to ophthalmic services and those utilization rates differences among various sociodemographic groups (11–13). Older age, female sex, and higher educational attainment groups statistically significantly associated with higher mean ophthalmic services expenditure in the United States (13)1,(11).

Health services utilization and OOP expenditure of ophthalmic services in developing countries are rarely studied before. We conducted a population-based survey to explore service utilization and OOP expenditure for blinding eye conditions and their determinants in Kurdistan Province.

## Materials and Methods

### Study area and sampling

Using a multi-stage population-based sampling, we conducted a study in Kurdistan Province- Northwest Iran in 2015. To compute a representative sample size we took into account the population of Kurdistan Province aged over 50 yr old (313665 persons), two-thirds of which living in 10 urban areas, the estimated prevalence of visual impairment (VI) in the Middle East region of 5.6%, a precision of 1, 10% non-response rate, a 95% confidence interval and a design effect of 1.5. In total, 3465 individuals from 99 clusters comprising 66 urban and 33 rural clusters of people 50 yr or older were surveyed. The clusters were selected through probability proportion to size sampling. In each cluster, samples were selected by a “cluster compact sampling” technique (14).

### Data collection

Ten trained teams comprising a community health worker and an optometrist reached the 10 districts and their randomly selected rural points, examined the eye health functions of participants, and interviewed them to complete the questionnaire including socio-demographic, eye and total health status, utilization and expenditure questions. Visual acuity was measured by optometrists at the household, using a Snellen tumbling “E” letter with optotype size 6/18 (20/60) on one side and 6/60 (20/200) on the other side at 6 and 3 m. Visual acuity was categorized according to WHO definition. Therefore, blindness was defined as visual acuity of <3/60, severe visual impairment as visual impairment ≥3/60 and <6/60 and moderate visual impairment as visual acuity≥6/60 and <6/18 in the better eye with available correction.

To assess the reliability and construct validity of the instrument we used the intraclass correlation coefficients, therefore a subset of 100 of the initial samples repeated the questionnaire within a 2-week period. To measure the ophthalmic and general health OOP expenditures we asked each participant total money they paid for eye and general health services during the recent last six months separately. General health care in this study defined as all of provided cares by Iranian health system. To estimate the utilization rate of ophthalmic services we obtained information on the frequency of visits the participants, had with ophthalmic services providers (ophthalmologist, optometrist or optician). To estimate general utilization rate, similarly, the total number of health services visits during the recent last 6-month was questioned. Measured social determinants of health were sex, educational level, age, ethnicity, career types, living place and economic status. To ascertain the latter, we asked the household monthly expenditure from suburban citizens and movable and immovable assets based on the residence. All expenditure data were converted from the Iranian Rial to the US dollar (32000Rial as average exchange rate in the year 2015). Before implementing the questionnaire, we pre-tested it to identify ambiguous wording, irrelevant categories, and awkward questions or questionnaire structure by conducting a focus group discussion containing related specialists such as an ophthalmologist, epidemiologist and health economist. Some of the categories were revised post hoc based on the observed frequencies.

### Statistical analysis

The expected yearly ophthalmic and total OOP expenditures were obtained by calculating the mean with its standard errors in various sociodemographic groups (15). All differences were considered statistically significant at P-value lower than 0.05 that corrected by Bonferroni method.

To avoid specification bias due to non-utilization with an ensuing zero OOP expenditure we employed a Heckman two-step regression model. In the first step, logistic regression was set up to predict decision on utilization using eye or total health status as predictors. In the next step, a linear regression model examined the impact of the sex, age, eye or general health status, ethnicity, education, career, living place and property as independent variables on expenditure. All statistical analyses were performed using Stata12 (StataCorpLP, College Station, Texas) statistical package.

### Ethical approval

This study was adherence to the guidelines of the Declaration of Helsinki and the ethical board of Tehran University of Medical Sciences and Iranian Ministry of Health approved the study proposal and we obtained a written free and informed consent from the participants.

## Results

From 3465 selected people in Kurdistan Province, 3240 (response rate: 93.5%) responded to the survey questions. The average age of the respondents was 62.7 (SD: ±10.09) yr with females comprising 51.9% of the sample with a domination of the Kurd ethnicity (95% of the sample). The average measure of intraclass correlation coefficients for the utilization and expenditure history instrument was calculated as 0.72.

### Eye services Utilization and OOP Expenditure

Table 1 summarizes self-reported utilization rates in the surveyed sample and by demographic variables. Statistically significant lower utilization rates were observed among men compared to women, middle-aged population compared to the elderly group, illiterate compared to the more educated participants, rural residents compared to urban dwellers, daily-paid workers compared to all other occupation categories, and the poorest participants compared to other income classes (*P*<0.001).

For 1188 (36.61%) who reported having an ophthalmic services visit in the last 6 months, mean OOP spending was US$43.7. People aged older than 65 yr paid statistically significantly higher OOP expenditure compared to those between 50 to 64 yr old. Other studied sociodemographic factors did not significantly impact OOP ophthalmic services expenditures, although spending was relatively higher for male, people with higher education level, Kurds, rural residence, self-employed subjects, and those with poor/near poor income status.

### Total Health services Utilization and OOP Expenditure

Of the 3240 respondents, 2620 (80.9%) reported having at least one health services visit during the last year. Daily-paid workers participants statistically significantly utilized lower health services compared with other occupation groups (*P*<0.05).

The utilization rates among other sociodemographic groups were not statistically different (Table 1).

**Table 1:** Average annual eye and total health care Out Of Pocket (OOP) in Kurdistan Province based on their socio-demographic characteristics

***Characteristics***	***N^a^ (%)***	***Eye Care OOP (in US Dollar)***			***Health Care OOP (in US Dollar)***			***Eye/Total (%)***

		***N^b^ (%)***	***Mean of OOP (SE)***	***P-value***	***N^c^ (%)***	***Mean of OOP (SE)***	***P-value***	
Total	3240 (100)	1185 (36.6)	43.7 (2.6)	-	2620 (80.9)	439.9 (22.8)	-	0.10
Sex								
Male	1558 (48.1)	512 (32.9)*	44.7 (4.2)	0.07	1157 (74.3)	420.2 (33.2)	0.41	0.11
Female	1682 (51.9)	673 (40.0)	42.7 (3.1)		1463 (87.0)	457.8(31.3)		0.09
Age groups								
50-64	2103 (64.9)	711 (33.8)*	30.7 (2.8)	<0.01	1672 (79.5)	414.7 (27.8)	0.13	0.07
65 and Over	1,137 (35.1)	474 (41.7)	67.7 (5.0)		948 (83.4)	486.0 (39.6)		0.14
Race								
Kurd	3,078 (95.0)	1137 (36.9)	44.8 (2.7)	0.06	2498 (81.2)	449.0 (23.8)	0.07	0.10
Non-Kurd	162 (5.0)	48 (29.6)	22.6 (5.1)		122 (75.3)	263.0 (58.4)		0.09
Education								
Illiterate	2,455 (70.85)	855 (34.8)*	47.1 (3.1)	0.07	2028 (82.6)	447.5 (24.8)	0.37	0.11
Less than High School	482 (13.91)	193 (40.0)	31.3 (5.1)		364 (75.5)	475.6 (81.8)		0.07
High School and greater	528 (15.24)	131 (24.8)	35.1 (6.2)		228 (0.43)	319.7 (51.2)		0.11
Living Place								
Urban	1,707 (52.7)	713 (41.8)	43.6 (3.5)	0.81	1405 (82.3)	471.8 (38.1)	0.26	0.09
Rural	1,119(34.5)	327 (29.2)*	45.2 (4.3)		880 (78.6)	417.8 (28.6)		0.11
Sub-urban	414 (12.8)	145 (35.0)	39.7 (7.7)		335 (80.9)	366.4 (32.0)		0.11
Occupation								
Unemployed	1895 (58.5)	749 (39.5)	46.5 (3.5)	0.28	1634 (86.2)	491.1 (33.2)	0.02	0.09
daily-paid workers	203 (6.3)	48 (23.6)*	30.8 (10.8)		131 (64.5)*	47.5 (34.4)		0.12
Permanently employed	218 (6.7)	92 (42.2)	31.7 (6.2)		163 (74.8)	408.2 (99.2)		0.08
Hired labor	52 (1.6)	21 (40.4)	20.1 (6.1)		36 (69.2)	146.1 (23.9)		0.14
Self-Employment	872 (26.9)	275 (31.5)	44.9 (5.0)		655 (75.1)	398.6 (35.3)		0.11
Property								
Poor/near poor	1,074 (33.1)	343 (31.9)*	40.7 (4.1)	0.81	802 (74.7)	433.7 (34.0)	0.97	0.09
Low income	1,391 (42.9)	546 (39.2)	44.3 (4.1)		7735 (77.3)	439.3 (36.4)		0.10
Mid/high income	775 (23.9)	296 (38.2)	44.4 (5.2)		569 (73.4)	448.1 (56.6)		0.10

a =Total number of participants;

b=Number of participants with ophthalmic care utilization;

c=Number of participants with healthcare utilization; OOP=Out-of-pocket; SE=Standard error; S=Not significant

* These sub-groups statistically significantly utilized lower eye and total health care compared with their counterparts

The self-reported mean OOP health services expenditure was $439.9 per year. These payments were not statistically significantly associated with any of the studied socio-demographic factors except occupation which unemployed participants statistically significantly paid higher OOP. However, results indicate higher expenditures among women, participants aged older than 65 yr, Kurds, those with less than high school education, urban residents and the mid-high income group (Table 1).

**Fig. 1:**
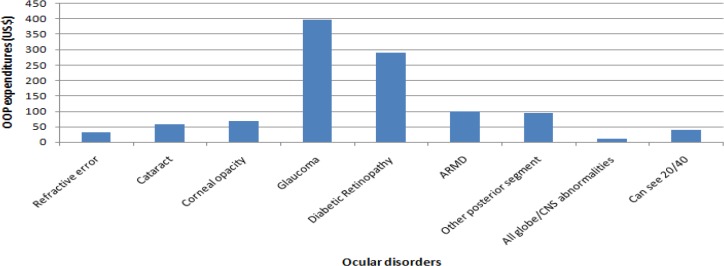
Mean of OOP expenditures by ocular disorders in Iranian population in 2015 Glaucoma and all globe/CNS abnormalities respectively had the highest and lowest out-of-pocket expenditure among Iranians with ocular disorders. ARMD: Age-Related Macular Degeneration; CNS: Central Nervous System

**Fig. 2:**
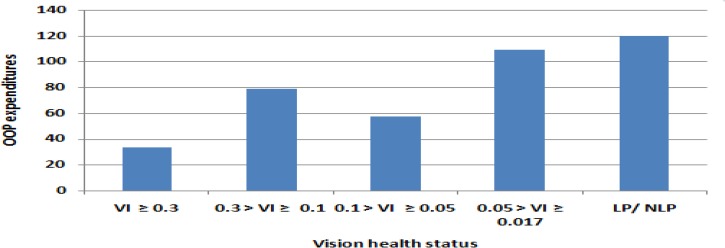
Mean of OOP expenditures by vision health status in Iranian population in 2015 The worsen vision health status, the higher ophthalmic out-of-pocket expenditure among Iranian population. VI: Visual Impairment; LP: light Perception; NLP: No Light Perception

Figures 1 and 2 show the utilization rate and mean of ocular OOP expenditure for a selected number of ophthalmic disorders and different levels of vision health status. Those patients with Glaucoma and the Diabetic Retinopathy disorders incurred the highest OOP expenditure, but in turn, had the most utilization rate compared with other ophthalmic disorders. The lowest ophthalmic OOP expenditure and utilization rate was related to all globe or CNS abnormalities. OOP expenditures show differing distribution across the eye conditions. The severity of the visual impairment was accompanied by higher utilization and OOP expenditure as well (Table 2).

**Table 2:** Annual utilization rate by blinding eye diseases (cutoff for visual impairment: a presenting VA of less than 20/40) and Vision Health Status

***Eye Diseases***		***Number (%)***	***Annual Utilization Rate (%)***
All		3237 (100)	1185 (36.6)
Refractive error		109 (3)	38 (34.9)
Cataract		158 (5)	56 (35.4)
Corneal opacity		37 (1)	16 (43.2)
Glaucoma		6 (0.2)	5 (83.3)
Diabetic Retinopathy		8 (0.2)	8 (100.0)
Age-related macular degeneration		61 (2)	31 (50.8)
Other posterior segment		22 (0.6)	10 (45.5)
All globe/CNS abnormalities		3 (0.1)	1 (33.3)
Can see 20/40		2477 (80)	1020 (36.0)
Vision Health Status	VI ≥ 0.3	2833 (88)	847 (34.2)
	0.3 > VI ≥ 0.1	390 (12)	162 (41.5)
	0.1 > VI ≥ 0.05	94 (3)	46 (41.9)
	0.05 > VI ≥ 0.017	98 (3)	53 (54.1)
	LP/NLP	48 (2)	26 (54.2)

VA=Visual Acuity; CNS=Central Nervous System; LP=Light Perception; NLP=No Light Perception Vision Health Status

Eye expenditure model indicates that OOP for ophthalmic services was positively and statistically significantly associated with females, people aged older than 65 yr, educated, daily paid workers and those with visual impairment variables compared with a reference group of each of this demographic variable.

This association was negatively and statistically significantly for rural, suburban dwellers, and unemployed factors. This model also shows significant relationship between probability of having OOP expenditure and income status or vision health.

Total health services expenditure model shows that the probability of total health services utilization as statistically significantly higher among females and lower among non-Kurd minorities and daily-paid workers. This Model does not show a statistically significant association between total health services utilization and other socio-economic status factors (Table 3).

**Table 3:** Multivariable Heckman two-step regression analysis of having out-of-pocket eye and healthcare expenditure by socioeconomic factors, 2015: The Kurdistan Eye Economics Survey

***Characteristics***	***Model 1: Eye Care Expenditure***			***Model 2: HealthCare Expenditure***		

	***Coefficient(SE)***	***95% CI***	***P-value***	***Coefficient (SE)***	***95% CI***	***P-value***
Sex						
Male	Reference^*^			Reference		
Female	0.30 (0.08)	0.15–0.45	<0.001	0.33 (0.09)	0.16–0.51	<0.001
Age groups (yr)						
50–64	Reference			Reference		
65 and older	0.25 (0.05)	0.15–0.36	<0.001	−0.04 (0.06)	− [0.16–0.08]	0.529
Race						
Kurd	Reference			Reference		
Non-Kurd	−0.11 (0.11)	− [0.33–0.10]	0.299	−0.29 (0.12)	− [0.52–0.05]	0.016
Education						
Illiterate	Reference			Reference		
<High School	0.28 (0.07)	0.13–0.42	<0.001	0.01 (0.08)	− [0.15–0.18]	0.862
High School	0.27 (0.10)	0.07−0.47	0.009	−0.07 (0.12)	− [0.30–0.16]	0.539
> High School	0.81 (0.21)	0.39–1.22	<0.001	0.07 (0.24)	− [0.40–0.53]	0.779
Area of Residence						
Urban	Reference			Reference		
Rural	−0.22 (0.07)	0.36–0.09	0.001	−0.05 (0.08)	− [0.20–0.11]	0.558
Suburban	−0.19 (0.09)	− [0.37–0.01]	0.038	−0.17 (0.10)	0.38–0.03	0.100
Occupation						
Unemployed	Reference			Reference		
Per diem laborer	−0.26 (0.13)	− (0.52–0.01)	0.044	−0.30 (0.13)	− (0.55–0.05)	0.019
Permanently employed	0.02 (0.12)	−0.23–0.26	0.883	0.01 (0.14)	−0.27–0.28	0.970
Employed laborer	0.14 (0.19)	−0.24–0.52	0.462	−0.14 (0.21)	−0.55–0.28	0.510
Self-employed	0.01 (0.08)	−0.16–0.17	0.951	0.04 (0.10)	−0.15–0.23	0.709
Income Status						
Poor/near poor	Reference			Reference		
Low income	0.05 (0.07)	−0.09–0.19	0.485	0.07 (0.08)	−0.09–0.23	0.371
Mid/high income	1.03 (0.10)	0.06–0.07	0.428	0.14(0.08)	−0.03–0.30	0.109
Visual Impairment						
None	Reference			Reference		
Mild	0.37 (0.10)	0.17–0.56	<0.001	0.05 (0.12)	−0.18–0.29	0.649
Severe	0.51 (0.19)	0.14–0.88	0.007	−0.23 (0.21)	−0.63–0.17	0.260

SE=Standard Error; CI=Confidence Interval //

* Model consider base variable as zero

## Discussion

Our findings indicate that in 2015 one-third of people over 50 yr old utilized an ophthalmic services service and 80% of them utilized a health service, in general, the average of vision and total health-related OOP expenditures among those using these services have estimated as US$43.7 and US$439.9, respectively. Australian people pay USD$1841 from their pocket over the year (12). The average of annual expenditure per ophthalmic patient older than 50 yr was estimated as about USD $2200 in the United States for 2015 (13). Even though eye disorders make up less than 1 percent of the total burden of all diseases in Iran (2), but ophthalmic disorders reconstituted about 10% of all health services OOP expenditures on average among individuals older than 50 yr. This ratio for American people was calculated about twice of our result (13). Current study is contrary to another study that shows the share of ophthalmic to total health OOP spending statistically significantly increasing with age. The major reason behind this positive association is that ocular disorders mostly are age-related disorders (3). This association did not see for total health services.

These results support the idea of those ophthalmic services are distributed unequally across different socio-demographic groups. Therefore, people, especially women, with lower socio-demographic status have utilized less ocular services compared with their counterparts that are accord with other related studies (16–18). Results concerning the inverse relationship between age and utilization rate were in line with provided in Australia (17) and in the United States studies (13). Labors paid the lowest OOP expenditure for eye and total health services compared with other occupation subgroups and not having a stable job leads to less expenditure. Theory of healthy worker effect appropriately explains this phenomenon that workers, in comparison to total population, are healthier, have less need for health services, and therefore pay lower OOP expenditure.

There is no significant relationship between visual impairment severity and OOP expenditure (12). This differs from the findings presented here and in another study (19). However, the presence of mild visual impairment, as compared to no visual impairment, was significantly associated with more ophthalmic expenditure, but this relationship was not seen in patients with severe visual impairment (13).

One important finding is that people with a vision of 20/40 and better-paid US $40.2 on average and one-third of them utilized the ocular services at least one time during the last six months. This utilization value is very close to the findings of Tehran Eye Study (20). This is despite the fact that persons with refractive error and central nervous system disorders with vision lower than 20/40, not only utilized the fewer ophthalmic services but also respectively with USD $30.4 and $10.4 had lower ocular OOP expenditure than healthy vision people that it’s reasons are not clear for authors due to no access to their insurance information. These patients had lower uptake and access to ophthalmic services and their health needs to glasses are not met completely.

For example, of 3 found patients with Central Nervous System, 2 cases reported no ophthalmic uses. Therefore, clearly, there is seen an unmet need in this patient’s group. For example, although blind persons used the eye health services as 1.6 times of those with healthy vision, paid 4 times of them and twice of cases with visual acuity between 0.05 and 0.1. About refractive error OOP expenditure, this OOP expenditure chronically paid and prorated over the life of these patients. Therefore, it is likely that we underestimated refractive error OOP expenditure and there is a need to longer time period analysis to extract values with lower uncertainty for this group.

Other ophthalmic disorders groups with vision less than 20/40 in compared with average values had a higher utilization rate and OOP payments. Among them, Cataract is almost a discrete event in one’s life and surgically treatable and appropriately reimbursed and patients with glaucoma and retinopathy respectively with USD $396.6 and $291.4 had the expected highest ocular OOP expenditure. These amounts are equal to the monthly income of an Iranian person (21). The reasons for this, on one hand, is that Glaucoma and Diabetic Retinopathy disorders are chronic problems that after diagnosis almost always require treatment and on another hand, their medicines and treatments such as Laser therapy and anti-vascular endothelial growth factor drugs injection are very costly that impose high ocular OOP expenditure on the patients (22). These findings are in agreement with those obtained by others (12). All found cases with diabetic retinopathy and 85% of patients with glaucoma in this study, were referred to ophthalmic providers and received eye health services.

The findings also show an uptake gap for those with age-related macular degeneration that seems resulted from its costly services. Therefore, regarding rising and aging population in next decades, health decision makers should prepare appropriate financial support to increase the access and minimize the existing unmet need.

### Study Strengths and Limitations

OOP-related findings must be interpreted with caution because the study is limited by few factors. Firstly, lack of access to health insurance data. Adjusting the findings with having or not have insurance coverage for participants may change our judgment on socio-demographic groups exposed to higher optical services costs. Secondly, current study potentially exposed to recall bias frequently inherited in retrospective designs. Although we asked the participants their ophthalmic OOP payments during the last 6 months in a separate question, it is expected that they, in addition to forgetting the exactly paid amount for ophthalmic services, also considered the OOP expenditures of other obtained health services in their reports. Thus, estimated eye-related OOP spending may suffer from overestimating. Thirdly, achieved low samples regarding people with Glaucoma, Diabetic retinopathy and visual impairment due to CNS disorders resulted in wider Standard Error in their statistics. Finally, due to no access to the medical records of participants, we could not decompose the components of payments such as visit, medicine, surgery, transfers and overhead costs. One of strengths of this study is that we examined the function of participant’s eyes to measure their eye health status, so this obtained data has high accuracy. Another one is generalizability of the results to Kurdistan people which arisen from study sampling method and sample size. Using Heckman two-step model also is the strength of this study. This model easy to implement and more efficient as long as is correctly specified (23). Throughout a system of equations, by a general-to-simple approach, this approach allowed us to model the amount of expenditure after considering the decision to utilize services.

## Conclusion

Ophthalmic diseases reconstituted about 10% of all health care OOP expenditures on average among individuals older than 50 yr. Our findings have important implications for developing eye health insurances. To decrease the eye OOP payments up to 50% among Kurdistan’s population with ocular disorders, health insurances, government or both of them totally should annually finance an additional 11.2 Million US dollar.

## Ethical considerations

Ethical issues (Including plagiarism, informed consent, misconduct, data fabrication and/or falsification, double publication and/or submission, redundancy, etc.) have been completely observed by the authors.
